# Retrotransposons in embryogenesis and neurodevelopment

**DOI:** 10.1042/BST20230757

**Published:** 2024-05-08

**Authors:** Mary Jo Talley, Michelle S. Longworth

**Affiliations:** 1 Department of Inflammation and Immunity, Lerner Research Institute, Cleveland Clinic, Cleveland, OH 44195, U.S.A.; 2 Cleveland Clinic Lerner College of Medicine, Case Western Reserve University School of Medicine, Cleveland, OH 44195, U.S.A.

**Keywords:** LINE-1, neurodevelopment, neurodevelopmental disorders, retrotransposon

## Abstract

Retrotransposable elements (RTEs) are genetic elements that can replicate and insert new copies into different genomic locations. RTEs have long been identified as ‘parasitic genes', as their mobilization can cause mutations, DNA damage, and inflammation. Interestingly, high levels of retrotransposon activation are observed in early embryogenesis and neurodevelopment, suggesting that RTEs may possess functional roles during these stages of development. Recent studies demonstrate that RTEs can function as transcriptional regulatory elements through mechanisms such as chromatin organization and noncoding RNAs. It is clear, however, that RTE expression and activity must be restrained at some level during development, since overactivation of RTEs during neurodevelopment is associated with several developmental disorders. Further investigation is needed to understand the importance of RTE expression and activity during neurodevelopment and the balance between RTE-regulated development and RTE-mediated pathogenesis.

## Introduction

Transposons, or transposable elements (TEs), are repeat elements that comprise almost half of the human genome [1]. TEs are genomic elements that have (or once had) the ability to move to new locations in the genome. DNA TEs, which move through a ‘cut-and-paste' mechanism, have mutated and lost the ability to transpose in the human genome [1]. Retrotransposons, or retrotransposable elements (RTEs), are genetic elements that undergo retrotransposition by replicating and inserting into the genome via an RNA intermediate, in a ‘copy-and-paste' manner.

RTEs are broadly categorized by the presence or absence of long terminal repeats (LTRs) that flank the RTE sequence. LTR RTE sequences closely resemble retroviral genomes, with LTRs flanking *gag* and *pol* genes. Some LTR RTEs also contain a functional *env* gene that may allow the RTEs to travel to surrounding cells [2–4].

**Figure 1.**
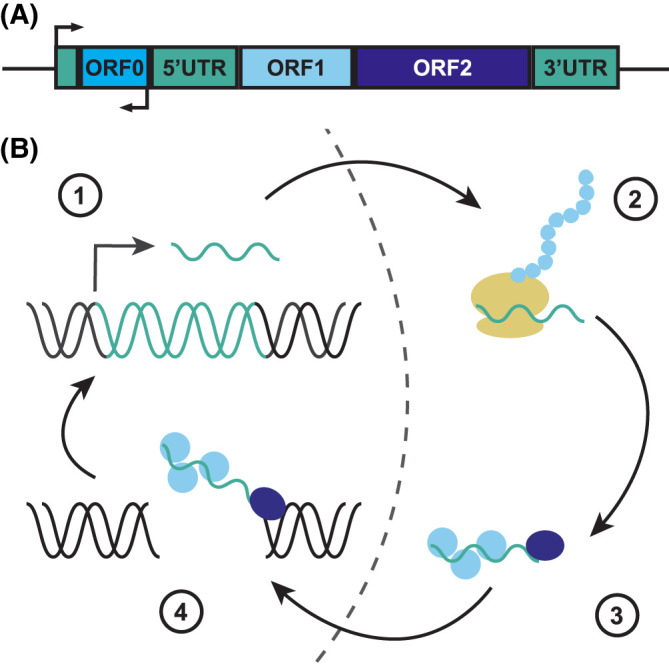
Lifecycle of an active long interspersed nuclear element-1 (LINE-1) element. (**A**) Structure of LINE-1. ORF1 encodes an RNA-binding protein that demonstrates nucleic acid chaperone activity while ORF2 encodes a 150 kDa protein that has endonuclease and reverse transcriptase capabilities [124–127]. Expression of both ORF1 and ORF2 is required for retrotransposition [128–131]. ORF0 is a primate-specific antisense reading frame located in the 5'UTR that may play a role in LINE-1 retrotransposition [5]. ORF0 also provides an alternative promoter in the antisense direction that may produce chimeric LINE-1 transcripts that may act as lncRNAs [60]. (**B**) Mobilization of LINE-1. (1) LINE-1 RNA is expressed and transported out of the nucleus. (2) LINE-1 RNA is translated. (3) ORF1 and ORF2 proteins preferentially bind to their own LINE-1 mRNA to form a ribonucleoprotein and transported back into the nucleus [125,130,132–134]. (4) ORF2 protein cleaves DNA and reverse transcribes LINE-1 back into the genome via target-primed reverse transcription (TPRT) [135–137]. TPRT is a process where a 3′-hydroxyl group that is revealed when ORF2p cleaves the genomic DNA is used as a primer to reverse transcribe LINE-1 mRNA. However, this classic understanding of reverse transcription is being challenged as evidence of reverse transcription has also been observed in the cytoplasm [18,20,111,137].

Conversely, non-LTR RTEs do not possess LTR sequences. The two major non-LTR RTEs are long interspersed nuclear elements (LINEs) and short interspersed nuclear elements (SINEs). LINEs are composed of two open reading frames (Figure 1). ORF1 encodes an RNA-binding protein that self-associates with the LINE mRNA. ORF2 encodes a reverse transcriptase and an endonuclease that promote insertion of LINE back into the genome. LINE-1 is the only autonomously active RTE in the human genome. However, while many LINE-1 loci are competent to retrotranspose, only a few elements are active [5–7]. An additional ORF, referred to as ORF0, has also been identified in the 5′UTR of LINE-1 [8]. ORF0 is a primate-specific open reading frame in the antisense orientation. ORF0 contains two splice donor sites and can form fusion proteins with proximal exons [8]. SINEs are short repetitive sequences and require LINE machinery to retrotranspose [9]. In humans, the most common SINEs are Alu elements. In addition to LINE & SINE, SINE-VNTR-Alus (SVAs) are hominid-specific non-LTR RTEs that also require LINE-1 reverse transcriptase to mobilize (reviewed in [10]).

RTEs can be a source of genomic instability with insertions causing developmental disorders and cancers (reviewed in [11–15]). Even without successful insertions, RTEs can increase DNA damage (reviewed in [16,17]). RTEs may also be expressed without successfully retrotransposing into the genome. RTE expression can induce inflammation, and emerging evidence suggests associations with many degenerative diseases, cancers, and general aging in several organisms ([4,18–32], reviewed in [33,34]). Due to the detrimental effects that RTEs can have on cells, many cellular mechanisms exist to inhibit RTE expression and mobilization. These mechanisms include transcriptional regulation controlled by repressive histone modifications and DNA methylation, as well as post-transcriptional regulation, including innate immunity and anti-viral pathways (reviewed in [10,13]).

## Retrotransposon activity during development

Despite the abundance of mechanisms that repress RTE activity, high levels of RTE expression have been observed during critical stages of development. RTE transcripts and proteins are highly expressed during early embryogenesis and during neurodevelopment. In this review, we compare and contrast the effects of RTE expression and activity in both embryogenesis and neurogenesis and discuss potential functional roles for RTEs during normal development.

### RTE expression and activity during embryogenesis

Retrotransposon activity is observed in all mammalian preimplantation embryos (reviewed in [35]). While the particular RTE families that are expressed and the timing of expression varies among species, it typically coincides with zygotic genome activation (ZGA) and decreases once the embryo reaches the blastocyst stage (reviewed in [35]). ERVs are expressed at varying levels in human embryogenesis; this expression is promoted by Dux in mice or DUX4 in humans, which are transcription factors that drive ZGA [36–45]. Embryonic stem cells (ESCs) and induced pluripotent stem cells (iPSCs) also show high LINE-1 expression [39,40,42,45–49].

To investigate the mobilization of LINE-1 in embryogenesis, several studies used a LINE-1 retrotransposition reporter. This reporter encodes an EGFP gene under the control of a CMV promoter positioned in the opposite orientation to the human LINE-1 transcript and interrupted by an intron. EGFP expression will occur in cells where LINE-1 has been expressed, spliced, and integrated into the genome [50,51]. Using this tool, LINE-1 was found to actively mobilize during embryogenesis [46,52]. Additionally, retrotransposition assays showed LINE-1 was 10-fold more efficient at mobilizing in human iPSCs than in the parental cell line [47]. Expression and mobilization of LINE-1 were also demonstrated in mouse iPSCs [53]. Next-generation sequencing (NGS) also confirmed retrotransposition events in iPSCs and ESCs [54,55].

### RTE expression in neurodevelopment

**Figure 2.**
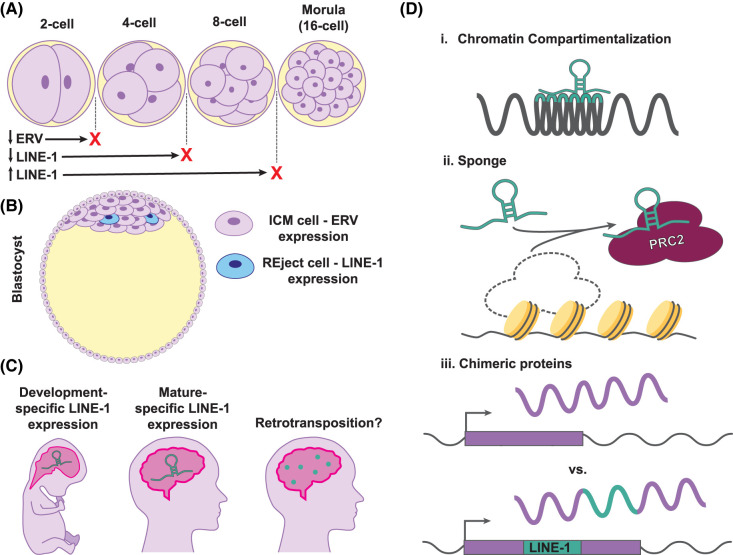
Summary of RTE activity in development. (**A**) During early embryogenesis, RTEs are expressed and required for development. Knockdown of MERVL halts development at the 2-cell stage, while depletion of LINE-1 halts development at the 4- to 8-cell stage during mouse embryogenesis [78–84]. Additionally, overexpression of LINE-1 also halts development at the 8- to 16-cell stage [84]. (**B**) After cleavage ends, different RTEs play different roles in development. In the human blastocyst, most cells within the inner cell mass (ICM) express HERV-H, which inhibits LINE-1 [39]. Cells that express LINE-1 also express DNA damage response markers and apoptotic genes; these cells are called ‘Reject cells' [39]. Reject cells are thought to be part of a ‘quality control' pruning process in the blastocyst [39]. (**C**) LINE-1 expression has been observed in both developing and aged human brains, where specific LINE-1 loci are differentially expressed [60]. While LINE-1 expression in the brain is supported by several studies, the mobilization rate of LINE-1 is currently disputed. (**D**) Mechanisms by which LINE-1 expression can impact development. (i) LINE-1 and Alu promote heterochromatin and euchromatin compartmentalization, respectively [87]. (ii) LINE-1 acts as a decoy for PRC2 in depositing repressive histone marks [59]. (iii) LINE-1 insertions can create chimeric proteins. These newly generated proteins may contribute to diversity in the brain (reviewed in [122]).

In 2005, Muotri et al. [56] observed LINE-1 expression in differentiating rat neural progenitor cells using microarray analyses. Subsequently, multiple reports confirmed LINE-1 expression throughout neural development [57–60]. Due to abundant LINE-1 sequences found within intronic DNA, care needs to be taken in RNA-seq experiments to not erroneously assign LINE-1 sequences present in another gene's intron as independent LINE-1 sequences, as these sequences are ultimately spliced out [61]. Recently, LINE-1 expression was carefully examined using a combination of RNA-seq and CUT&RUN to identify chromatin enriched for H3K4me3 to identify putative active promoters [60]. This allowed individual LINE-1 elements to be aligned to specific loci within the genome [60]. Single cell and bulk RNA-seq of human brain samples from both adults, aged 69–87, and from fetuses, aged 7.5–10 weeks, showed high expression of LINE-1 in both developed and developing brains. By clustering single-cell RNA-seq data and backtracing, authors found LINE-1 expression to be enriched in neurons in adult samples, and little to no expression in glial cells. In embryos, LINE-1 was highly expressed in apical and basal progenitors, as well as early born neurons. Interestingly, the LINE-1 transcripts expressed in fetuses and adults were from different loci (Figure 2C). The authors hypothesized these differences were likely due to different chromatin landscapes, as loci containing LINE-1 expressed during development were often located in introns of genes that exhibited developmental-specific expression patterns [60]. Alu elements have also been identified in neurodevelopment and can greatly impact genomic landscape and gene expression [62]. LTR RTEs expression during neural development is not well understood. A study of HML-2, a HERV-K subtype, determined that HML-2 env transcripts are down-regulated from iPSCs differentiating into neural lineages, and that expression may, in fact, antagonize to neural development [63]. However, a recent publication demonstrated expression of RNLTR12-int, an endogenous retrovirus, regulates the production of Myelin Basic Protein in oligodendrocytes during developmental myelination [64]. Further work is required to understand the regulation of RNLTR2-int in oligodendrocyte development and to determine whether other LTR might also regulate the development of neural cell types.

The increased expression of RTEs in the brain is due, at least in part, to relaxed transcriptional inhibition. LINE-1 contains SOX binding sites within its promoter, where SOX transcription factors can bind and repress expression [65]. During brain development, LINE-1 was demonstrated to be repressed by Sox2 binding [56]. During neuronal differentiation, Sox2 expression and binding to the LINE-1 5′UTR decreased, as LINE-1 expression increased [56]. The removal of Sox2 binding, including the binding to LINE-1 5′UTR, is associated with Wnt/β-catenin activation during brain maturation [66]. As Sox2 repression of LINE-1 is reduced, global H3K9 methylation and histone deacetylase I (HDAC1) binding is also decreased in the differentiating neural precursors, allowing for increased accessibility of LINE-1 containing chromatin [56].

### Retrotransposition during neurodevelopment

While current literature is generally in agreement that LINE-1 is highly expressed during neurodevelopment, the mobilization rate of LINE-1 during neurodevelopment remains questionable. Reports differ in their estimates of retrotransposition, with a large range starting from 0.04 retrotransposition events per cell up to 80 events per cell [58,67]. This range is likely due to the use of different technology and methods to identify true retrotransposition events. Transgenic mice expressing the LINE-1 retroposition reporter, described above, showed EGFP expression throughout the brain, but only in neurons (as indicated by neuronal marker NeuN) and not in glial cells [56]. *In vitro*, both neural precursor cells (NPCs) and mature neurons exhibited EGFP expression, indicating both progenitors and mature cell types can accommodate LINE-1 retrotransposition [56–58]. Interestingly, this exogenous LINE-1 cassette did not retrotranspose in other somatic tissues, suggesting neurons may be unique among somatic tissues in their competency for retrotransposition [56–58]. While these studies have shown the ability of LINE-1 to retrotranspose in NPCs and neurons, it is possible that endogenous LINE-1 elements could behave differently in these cell types.

In addition to the LINE-1 reporter, NGS is commonly used to identify retrotransposition events [67–72]. While NGS provides valuable information about endogenous LINE-1 mobilization, differences in library preparation and sequencing technology used in different studies have likely led to vastly different results (reviewed in [73]). One experimental method that has produced results which support the hypothesis that high levels of LINE-1 retrotransposition occur in neurons was retrotransposon capture sequencing, referred to as ‘RC-seq', in which custom tiling arrays with probes against the 5′ and 3′ termini of LINE-1 were used to hybridize DNA [68–70]. This produced estimates between 13.7 insertions per neuron within the hippocampus to 0.2 retrotransposition events per cell, depending on the analysis done [69,70]. Another experimental technique, SLAV (somatic LINE-1-associated variants) -seq' was used to investigate LINE-1 retrotransposition rates in whole NeuN+ single nuclei genomes from post-mortem hippocampi and frontal cortices [71]. This uncovered frequency ranges from 0.75 to 1.5. Single nucleotide sequencing of NeuN+ neurons estimated a rate of 0.04–0.6 insertions per neuron [71]. A study investigating the rate of somatic transposition in *Drosophila* found that library preparation can lead to artifacts that can falsely be aligned to RTEs, suggesting the RTE insertions detected in previous studies, including within the Drosophila brain, may be overstated [74,75]. Most recently, single-cell whole genome sequencing, RC-seq, and L1 insertion profiling of hippocampal neurons were combined, providing the most comprehensive understanding of LINE-1 retrotransposition rate, which suggested frequencies of <1 event per cell, similar to previously described frequencies [67,71,72,76]. However, it was noted that with the current technology, it is difficult to obtain a truly accurate rate of mobilization. Although the rate of retrotransposition in neuronal lineage cells is currently disputed, even low rates of retrotransposition can have large impacts on how the brain functions, as suggested by Faulkner and Billon in a previous review [77].

## Consequences of retrotransposon activity during development

### Requirements for RTEs in embryogenesis

RTE activity has been identified as an integral part of early embryogenesis. Knockdown of mouse ERV-L (MERV-L) induces developmental arrest of embryos before the blastocyst stage [78–82]. One study used siRNA, three independent antisense oligonucleotides, CasRX, and CRISPRi to show comprehensively that MERV-L depletion halts development (Figure 2A) [82,83]. Interestingly, it was shown that MERV-L RNA, but not the mRNA or protein products, were required for proper development, which the authors suggest may be due to MERV-L transcripts acting in *cis* to regulate gene expression [82].

LINE-1 also plays an important roles in embryonic development (Figure 2A) [84]. LINE-1 is highly expressed in the early stages of mouse preimplantation development, with the highest expression at the 2-cell stage and declining through the 8-cell stage. To manipulate endogenous LINE-1 expression, one study used transcription-activator-like effectors (TALEs) bound to VP64 and KRAB to overexpress and repress endogenous LINE-1 at the late 2-cell stage, respectively. Prolonging LINE-1 activity beyond the 2-cell stage with TALE-VP64 led to lower rates of development, stopping development between the 8-cell and 16-cell stages. Overexpressing LINE-1 in the presence of Zidovudine, a nucleoside reverse transcriptase inhibitor (NRTI) that prevents LINE-1 retrotransposition, did not improve the developmental rate, suggesting that it is the expression of LINE-1, and not the retrotransposition activity, that impacts developmental timing. Repression of LINE-1 with TALE-KRAB also had adverse effects, halting development between the 4-cell or 8-cell stage. Global transcription was reduced following LINE-1 repression, and LINE-1 was suggested to modulate global chromatin accessibility, including both decondensation and recondensation [84]. Additionally, LINE-1 was shown to act as a nuclear RNA scaffold that represses Dux at the blastocyst stage to repress 2-cell-specific transcriptional profiling, further supporting the role of LINE-1 in embryonic development [49].

Additional evidence suggests that the ability of RTEs to modulate chromatin organization may be paramount for their roles during embryogenesis (Figure 2D). RTE transcripts have been associated with chromatin organization and topologically associating domains (TADs). HERV-H RNA associates with chromatin, aiding in TAD boundary organization [85,86]. LINE-1 and Alu (called B1 in mice) also impact 3D genome organization [87,88]. LINE-1 was suggested to promote heterochromatin compartmentalization into B compartments in ESCs, and B1 RTEs possess similar functions to promote euchromatin A compartments [87]. This organization seems to rely on LINE-1 expression, as depletion of LINE-1 RNA drastically altered global chromatin structure [87]. Zidovudine treatment did not impact this activity, once again suggesting expression, but not mobilization, is important for LINE-1-associated functions during development. Alu RNA has also been shown to promote enhancer–promoter looping [88].

Slightly later in development, HERV-H and LINE-1 expression was associated with different cell types in the inner cell mass (ICM), the part of the blastocyst that will develop into the fetus (Figure 2B). High LINE-1 expression was associated with ‘REject cells', previously uncharacterized cells that also express DNA damage response markers and apoptotic genes [39]. HERV-H was associated with surviving cell types and suppressed LINE-1 expression [39]. Authors hypothesized RTEs may possess ‘quality control' functions to eliminate cells that have undergone damage or to prune cells that are no longer needed.

Together, these studies suggest that RTEs may be important for regulating global chromatin organization and gene expression during early embryogenesis, and they may be more important for pruning damaged or unwanted cells during later embryogenesis.

### Requirements of RTEs in neurodevelopment

Little is known about the role of RTEs in neurodevelopment. Recently, a short-hairpin RNA (shRNA) was designed to target a conserved region of LINE-1 (referred to as shL1) [59]. This shL1 or a control scrambled shRNA, along with a GFP reporter, was electroporated into mouse embryo cortices. GFP expression in the mice suggested disturbances in differentiation, cell fate decisions, and neuron migration in cells expressing shL1 versus control shRNA. RNA-seq was performed in embryos expressing shL1 or control shRNA, as well as *in vitro* neuronal cultures expressing the shRNAs. Knockdown of LINE-1 *in vivo* led to the down-regulation of genes associated with cell metabolism and the up-regulation of genes associated with neurodevelopment. However, the opposite effects on gene regulation were observed in *in vitro* experiments*.* The authors suggested that the differences between *in vivo* and *in vitro* results were due to different developmental time points observed in the two systems, and further suggested that LINE-1 is required and plays different roles throughout development. Interestingly, wild-type neural cultures treated with NRTIs showed very little differential gene expression, suggesting retrotransposition has little impact on gene expression during neurodevelopment, lending support to the idea that a non-retrotransposition-independent role of LINE-1 is important for regulating neurodevelopment [59]. LINE-1 transcripts were recently discovered to physically associate with repressive chromatin. ChIP-seq experiments demonstrated that LINE-1 RNA influences polycomb repressive complex 2 (PRC2)-dependent deposition of repressive histone modifications in genes controlling neurodevelopment, potentially acting as a sponge or decoy for PRC2 (Figure 2D) [59]. While it was recently revealed that artifacts can be generated when depleting RNA during ChIP-seq, resulting in increased noise which impacts the analysis of PRC2 binding [89–91], the experimental design between these studies and the LINE-1 study differ in a way that likely avoided this potential artifact. Further investigation of the PRC2 and LINE-1 interaction will be required to determine whether the functional significance is conserved in human neurons.

Additional studies suggest LINE-1 may possess a retrotransposition-independent role in regulating NPC differentiation. ShRNA-mediated depletion of active LINE-1 families *in vitro* and *in vivo*, in mice and human cerebral organoid cultures, induced precocious differentiation of NPCs [92]. This effect was due to LINE-1 expression, as the reintroduction of full-length, wild-type LINE-1 or LINE-1 encoding a mutated reverse transcriptase both rescued the neural differentiation [92]. Furthermore, human-specific LINE-1-derived long noncoding RNA (lncRNA), *LINC01876*, may also significantly impact NPC differentiation [60]. CRISPRi-mediated silencing of this specific lncRNA resulted in premature differentiation of NPCs and subsequently smaller cerebral organoids. Finally, a LINE-1 insertion was shown to be beneficial to parvalbumin-positive (PV+) interneuron development by functioning as a cis-regulatory element that promotes transcription of PV+ genes that influence neuron morphology and function, thus uncovering a potential positive effect of LINE-1 induced somatic mosaicism [93]. Together, these findings suggest that LINE-1-derived RNAs provide a layer of genetic complexity to brain development, either through function as lncRNA, through the promotion of heterochromatin formation, and/or through the regulation of gene expression.

### Increased RTE activity is associated with neurodevelopmental disorders

Excessive RTE activity has been associated with several neurodevelopmental disorders. RTE insertions have been identified as, or have been putatively associated with, the mutagenic causes of neurodevelopmental disorders, including Nf1 where 0.4% of all mutations are predicted to be from RTE insertions [94–98]. Ataxia–Telangiectasia, Autism Spectrum Disorder (ASD), Rett Syndrome, Alpha Thalassemia X-linked Intellectual Disability Syndrome, Schizophrenia, and other neurological and neurodegenerative diseases have been associated with increased RTE and LINE-1 expression and activity ([99–108], reviewed in [12]). Human active transposon sequencing (HAT-seq) analyses identified L1Hs insertions in both brain and non-brain tissues of Rett syndrome patients, suggesting the insertions occurred during embryogenesis in these individuals [103]. Furthermore, clonal somatic insertions were enriched in introns, in the sense of orientation to transcripts, and the authors hypothesized that this could affect the transcriptional elongation of neighboring genes, although direct evidence was not presented. An argument against LINE-1 insertion-mediated disease pathogenesis was presented in recent experiments that re-expressed MECP2 in MECP2-depleted mouse models of Rett syndrome, suggesting the physical insertion of LINE-1 was not causative [109].

The current links between RTEs and ASD are largely correlative. A recent study comparing whole genome sequences obtained from PBMCs of dizygotic twins with non-syndromic autism demonstrated unique, *de novo* Alu insertions enriched in active enhancers in ESC-neurons, although insertion-mediated transcriptional deregulation was not experimentally tested [98]. Additionally, a re-analysis of RNA-seq data from prefrontal cortex tissues of ASD patients and matched controls showed increased Alu element expression in ASD patients, with correlation analyses predicting Alu-mediated dysregulation of genes involved in neuronal cell cycle, cell death and neuroinflammation [107]. Finally, a re-analysis of RNA-seq data from postmortem brain tissue of ASD patients, cultured cells depleted for ASD-associated genes, and blood of discordant siblings detected up-regulation of young LINE-1 expression, primarily located within introns of actively transcribed genes [104]. These genes were involved in the regulation of synapse organization and function, and their expression was negatively correlated with LINE-1 expression. In all of these ASD-focused studies, the correlations between increases in RTEs and differential gene expression were not experimentally validated. Furthermore, the majority of these studies were conducted NGS techniques which, as discussed earlier, can be difficult to interpret and are prone to artifacts. Future studies will need to confirm RTE activity in these diseases, as well as identify causative roles for RTEs.

Several elegant studies using patient cells and patient-derived organoids investigating elevated LINE-1 expression and activity in Aicardi–Goutières syndrome (AGS) have contributed to a mechanistic understanding of how LINE-1 may promote neurodevelopmental disorders. AGS is an inflammatory neurodevelopmental disorder that causes severe physical and intellectual impairments. Genetic mutations causing AGS are associated with anti-viral responses, including mutations in 3′ repair exonuclease 1 (Trex1). These mutations lead to the accumulation of single-stranded DNA (ssDNA) derived from RTEs [110]. TREX1-deficient neural cells accumulate ssDNA, mostly originating from LINE-1. Neurons derived from TREX-1 deficient iPSCs exhibited increased apoptosis and cerebral organoids generated from iPSCs were smaller than controls. TREX-1 deficient astrocytes secrete type I interferons. Treatment with NRTIs that prevent LINE-1 RT rescued these phenotypes [111]. NRTIs were also shown to ameliorate the myocarditis in Trex1-null mice [112]. One study failed to replicate these findings using a different set of reverse transcriptase inhibitors [113]. However, it should be noted that NRTIs were generated as therapeutics for HIV and, while the authors showed these drugs do reduce mobilization of LINE-1, future studies would need to confirm that the NRTIs used in this study prevent the generation of LINE-1 ssDNA as well [32,113,114]. These results suggest that the immune response prompted by LINE-1 ssDNA may be responsible for the phenotypes observed in AGS. Another gene mutated in AGS, RNase H2, is an anti-viral protein that degrades the RNA in RNA/DNA hybrids (reviewed in [115]). A knock-in mouse model with the human RNAse H2 mutation also exhibited LINE-1 DNA in the cytosol, but ORF1 protein was not increased. Interestingly, knocking out Sting, a component of the innate immune system that promotes expression of inflammatory genes in response to cytosolic DNA, did not decrease LINE-1 DNA in the cytosol, but did partially rescue the lethality of the RNAse H2 mutant knock-in mice [116]. Mutations in the retroviral restriction enzyme, SAMDH1, can also cause AGS [117,118]. SAMHD1 was also found to antagonize LINE-1 expression and reverse transcription [119]. While more research needs to be conducted to understand the impact of LINE-1 expression and activity on pathogenesis associated with neurodevelopment, the progress made in AGS research suggests that LINE-1-mediated activation of the innate immune system could be responsible for many of the phenotypes. Interestingly, RTEs were also recently demonstrated to play a causative role in *Drosophila* models of primary microcephaly [120]. In these condensin insufficient models of microcephaly, RTE expression and mobilization caused cell death in the developing brain, leading to smaller head sizes, and this could be rescued by allowing *Drosophila* to develop on food containing NRTIs [120].

Finally, machine learning-facilitated investigation into LINE-1 somatic insertions in neurons and glia from adult brains of schizophrenia patients and matched controls, in comparison with fetal brains, identified two LINE-1 insertions in neuropathology-associated genes that likely occurred in neuroepithelial cells that give rise to neural stem cells [121]. Excitingly, the authors cloned these LINE-1 sequences into reporter constructs and demonstrated reduced reporter expression, suggesting the insertions have the potential to negatively affect the transcription of the neuronal genes into which they are inserted.

## Conclusion

While high RTE activity may seem to be unfavorable to cellular fitness, recent research is supportive that this activity is beneficial to normal embryogenesis and neurogenesis. Previously it has been suggested that RTE activation during neurodevelopment leads to beneficial somatic mosaicism, and that RTE insertions potentially contribute to new, diverse genomes within individual neurons [122]. However, evidence for this beneficial somatic mosaicism is limited and RTE activity could just as easily lead to detrimental mutations [93]. Recent studies in both embryogenesis and neurodevelopment suggest that RTE expression may influence gene expression. However, in both systems, retrotransposition activity is not required. Additionally, studies of pregnant women prescribed anti-viral medications, which would prevent retrotransposition of RTEs, have not found any negative effects to the embryo's development (reviewed in [123]). If the expression of RTEs, and not the mobilization, is important for development, then in diseases like AGS, where RTE mobilization may contribute to pathogenesis, NRTIs and other anti-viral medications that block retrotransposition could be beneficial to reverse or inhibit further pathogenesis caused by RTE mobilization, without disrupting normal development. Indeed, clinical trials are currently ongoing to test whether NRTIs can treat AGS (https://clinicaltrials.gov/study/NCT02363452). Continued investigation into the role of RTEs in neurodevelopment and any gene regulatory functions they serve will provide beneficial information for treating some neurodevelopmental disorders.

## Perspectives

- Retrotransposon activation during embryogenesis and neurodevelopment is a poorly understood phenomenon.
- Current research suggests that retrotransposon RNA may play a role in chromatin organization and gene regulation.
- Further research needs to be done to understand whether retrotransposition plays a role in development and to better understand the differences that may distinguish the benign effects of RTE activation during normal development from the pathogenic effects of RTE activation that occur in neurodevelopmental disorders.

## Open Access

Open access for this article was enabled by the participation of Cleveland Clinic in an all-inclusive *Read & Publish* agreement with Portland Press and the Biochemical Society under a transformative agreement with Individual.

## Abbreviations

AGS: Aicardi–Goutières syndrome
ESCs: embryonic stem cells
iPSCs: induced pluripotent stem cells
LINEs: long interspersed nuclear elements
lncRNA: long noncoding RNA
LTRs: long terminal repeats
MERV-L: mouse ERV-L
NGS: next-generation sequencing
NPCs: neural precursor cells
NRTI: nucleoside reverse transcriptase inhibitor
PRC2: polycomb repressive complex 2
PV+: parvalbumin-positive
RTE: retrotransposable elements
RTEs: retrotransposable elements
shRNA: short-hairpin RNA
SINEs: short interspersed nuclear elements
ssDNA: single-stranded DNA
TEs: transposable elements
ZGA: zygotic genome activation
